# Lower-Risk Myelodysplastic Syndrome (MDS) Patients Exhibit Diminished Proteasome Proteolytic Activity and High Intracellular Reactive Oxygen Species (ROS) Levels

**DOI:** 10.7759/cureus.49843

**Published:** 2023-12-02

**Authors:** Kalliopi Zafeiropoulou, Georgios Kalampounias, Spyridon Alexis, Theodosia Androutsopoulou, Panagiotis Katsoris, Argiris Symeonidis

**Affiliations:** 1 Department of Biology, University of Patras, Patras, GRC; 2 School of Medicine, University of Patras, Patras, GRC; 3 Hematology Division, Department of Internal Medicine, University General Hospital of Patras, Patras, GRC

**Keywords:** ubiquitin-proteasome system, cd34 positive, bone marrow, oxidative stress, bortezomib, acute myeloid leukemia (aml), reactive oxygen species, ipss-r, proteasome inhibitors, myelodysplastic syndromes

## Abstract

Myelodysplastic syndromes (MDS) constitute a heterogeneous group of clonal hematopoietic stem cell disorders characterized by ineffective hematopoiesis and an elevated risk of transformation to acute myeloid leukemia (AML). Available disease-modifying treatment approaches are limited. The ineffectiveness of proteasome inhibitors (PIs) in MDS patients is currently investigated, although it is unclear whether they rapidly develop resistance to PIs or whether proteasome proteolytic activity (PPA) is constitutively lower in the hematopoietic cells of these patients, thus limiting treatment effectiveness. We investigated 20 patients with MDS, categorized according to the International Prognostic Scoring System (IPSS) into a lower- or a higher-risk group. Peripheral blood mononuclear cells, bone marrow mononuclear cells, and cluster of differentiation 34-positive (CD34+) cells were isolated and assessed for the chymotrypsin-like activity of the proteasome and β5 subunit accumulation. Additionally, intracellular reactive oxygen species (ROS) generation was screened. The lower-risk patient group (n=10) exhibited significantly lower proteasome activity (p<0.001) compared to both the higher-risk group (n=10) and healthy subjects (n=10). Furthermore, the lower-risk group had elevated oxidative stress levels (p<0.0001) and reduced β5 subunit expression (p=0.0286). Both parameters were shown to be associated with transfusion dependency, since transfusion-dependent patients (n=5 in each subgroup) had decreased proteasome activity and simultaneously exhibited higher ROS levels. Our results indicate that reduced β5 expression might potentially explain PIs' ineffectiveness in lower-risk MDS, elucidating the importance of the risk group in the selection of the proper treatment algorithm.

## Introduction

The proteasome is a multicatalytic intracellular protein complex that degrades by proteolysis many unneeded, misfolded, damaged, or simply excessive intracellular proteins in the cytoplasm and nucleus of all eukaryotic cells [1]. It is a multimolecular structure composed of proteinic complexes centered on the 20S proteasome core particle that houses three proteolytic sites in its hollow interior [2]. The 20S core particle has a molecular weight of approximately 700 kDa and consists of 28 protein subunits that are arranged in four homologous rings of seven subunits each, forming a hollow cylindrical structure reminiscent of a barrel. The two inner rings are formed by seven β subunits (β1-7) and are enclosed by the two outer rings, assembled from seven α subunits (α1-7) [3,4]. The central proteolytic chamber, formed by β rings, has three catalytically active subunits, exhibiting active forms of caspase-like (C-L) β1 subunit, trypsin-like (T-L) β2 subunit, and chymotrypsin-like (ChT-L) β5 subunit [5]. ChT-L activity plays a key role in the intracellular degradation of proteins, especially those determining tumor growth and survival [6].

Inhibition of proteasomal activity has been proven to be a major therapeutic target for both hematological malignancies and solid tumors [6]. Numerous clinical trials have demonstrated that proteasome inhibitors (PIs) are well-tolerated and highly effective agents, used alone or in combination with traditional chemotherapeutic or other targeting agents, aiming to enhance their therapeutic effect [7,8]. Until today, there is no clear evidence that PIs, such as bortezomib, could represent an effective treatment option for patients with myelodysplastic syndromes (MDS), a group of clonal hematopoietic stem cell disorders characterized by ineffective hematopoiesis and a high risk of transformation to acute myeloid leukemia (AML). For these diseases, the International Prognostic Scoring System (IPSS) and its revised version (IPSS-R) are useful tools commonly used to evaluate the risk of AML transformation and the overall survival of MDS. These simple algorithms combine the number and severity of the cytopenias, the percentage of bone marrow blasts, and marrow cytogenetics to classify MDS patients into four or five prognostic groups with different risks of progression to AML and different survival: low, intermediate-1 (int-1), intermediate-2 (int-2), and high risk for the IPSS and very low, low, intermediate, high, and very high risk for the IPSS-R [9].

Treatment options for higher-risk MDS (HR-MDS) patients, especially for the younger group (less than 65 years old), include allogeneic hematopoietic stem cell transplantation, various targeted types of treatment, and combination chemotherapy [10]. For elderly patients, ineligible for intensive approaches, treatment options are limited to hypomethylating agents (HMAs), such as azacitidine or decitabine, and an emerging group of newer agents (multikinase inhibitors, immune checkpoint inhibitors, anti-apoptotic inhibitors, and inflammation checkpoint inhibitors) used alone or in combination with HMAs [11], for which the clinical experience is still limited. Finally, for patients with lower-risk MDS (LR-MDS), mainly supportive types of treatment, such as red blood cell (RBC) transfusions, erythropoiesis-stimulating agents (ESA), lenalidomide, and iron chelation treatment (ICT), are usually administered. This policy aims to improve the quality of life of MDS patients, although it does not substantially affect AML transformation or modulate the natural history of the disease [12]. The need for the discovery of new targeted therapies for MDS patients is now more imperative than ever before. Indeed, in recent years, newer agents targeting erythroblastic apoptosis, such as luspatercept, or the intracellular oxidative environment, such as roxadustat, or inhibiting telomerase, such as imetelstat, have emerged, aiming to render erythropoiesis less ineffective [13-15]. However, clinical trial data have shown that PIs, such as bortezomib, have no significant effect on MDS, especially in lower- or intermediate-risk patients with severe cytopenias and multilineage dysplasia [16,17]. It is not yet clear whether these patients develop rapid resistance to PIs or whether proteasome proteolytic activity (PPA) in these patients is constitutively low [18], and therefore, they intrinsically exhibit primary resistance to PIs. Thus, it appears that this drug category is not effective for LR-MDS patients. Notably, potential insufficient PPA in the hematopoietic stem cells of MDS patients might induce the accumulation of oxidatively modified or mutated and misfolded proteins, which would further disrupt the cellular homeostasis of the dysplastic cells and potentially accelerate transformation to AML [19]. Thus, determination of PPA and endogenous reactive oxygen species (ROS) levels in the hematopoietic stem and progenitor cells of these patients could be a useful tool for predicting the effectiveness of treatment with a PI or the risk for AML transformation.

This study aimed to focus on the PPA of patients with MDS and to investigate whether there would be any association between the 26S proteasome function and the prognostic scores of these individuals. Parameters such as ChT-L activity (which is impaired by PIs) and the localization and accumulation of the proteasome subunit beta-5 (PSMB5) subunit (the main proteolytic subunit, possessing the former type of activity) were assessed. Given the fact that elevated oxidative stress is an AML progression marker [20], the generation of intracellular ROS was also assessed. Finally, a relation between the transfusion dependency and the former two markers was investigated, since blood transfusions have been correlated to increased blood contents of oxidized proteins, which induce responses related to oxidative stress, possibly also affecting the ubiquitin-proteasome system (UPS) [21].

## Materials and methods

Subject population

Patients with MDS (n=20), healthy blood donors (n=10), AML patients (n=10), and non-MDS subjects-volunteer hemopoietic stem cell donors (n=10) were recruited between January 2021 and August 2022. Twenty patients with newly diagnosed (within 100 days of diagnosis) MDS were grouped according to their IPSS score into a lower- or higher-risk group. The lower-risk group (LR-MDS, n=10) included patients with an IPSS score of 0-1, while the higher-risk group (HR-MDS, n=10) included patients with an IPSS score of ≥1.5. Peripheral blood from 10 healthy age-matched subjects and bone marrow aspirates from 10 non-MDS donors without infiltration were used as controls. In addition, peripheral blood and bone marrow samples from AML patients were used as positive controls for proteasome activity.

Sample collection

Peripheral blood mononuclear cells (PBMCs) were isolated from a 20 ml peripheral blood sample using Lymphosep™ density gradient medium (Cat. No. L0560, Biowest, Rue du Vieux Bourg, Nuaillé, France). The same protocol was used to isolate bone marrow mononuclear cells (BMMCs) from a 5 ml bone marrow aspiration sample. Cluster of differentiation 34-positive (CD34+) cells were isolated from BMMCs using magnetic-activated cell sorting (MACS, Miltenyi Biotec, Bergisch Gladbach, North Rhine-Westphalia, Germany) following the manufacturer's instructions. All patients were informed about the purpose of the study and signed informed consent before their participation. Ethics approval for all experimental protocols was granted by the University General Hospital of Patras Ethics Committee (approval number: 17955/20-07-2020).

Proteasome activity assay

Total proteins were extracted from the cells, using sonication and incubation in a buffer solution containing 50 mM 4-(2-hydroxyethyl)-1-piperazineethanesulfonic acid (HEPES), 20 mM potassium chloride (KCl), 5 mM magnesium chloride and water (MgCl_2_·H_2_O), and 1 mM dithiothreitol (DTT) (Cat. No. 10197777001, Merck, Darmstadt, Germany) with a pH value of 7.81. The extract was incubated with proteasome fluorogenic substrate peptide leucine-leucine-valine-tyrosine-7-amino-4-methylcoumarin (LLVY-AMC) (Cat. No. 3120-v, Peptide Institute Inc., Osaka, Japan), with or without the PI MG-132 (Cat. No. 3175-v, Peptide Institute Inc., Osaka, Japan) inside a flat-bottom black polystyrene 96-well plate for fluorometric assays for one hour at 37°C. Fluorescence intensity was measured at 380 nm excitation wavelength and 460 nm emission wavelength using a Tecan Magellan spectrofluorometer (Tecan, Männedorf, Switzerland). Total protein concentration was estimated using the Q5000 NanoDrop Quawell spectrophotometer (LabWrench, California, United States). The ChT-L activity results were normalized using total protein concentration and presented as fluorescence per mg of total protein.

Western blot analysis

Cells were lysed, using radioimmunoprecipitation assay (RIPA) buffer supplemented with phosphatase and protease inhibitors, and the cell contents were homogenized using sonication. Total protein concentration was estimated with the Bradford assay. Equal amounts of total proteins were mixed with Laemmli's sample buffer 2X solution containing 5% 2-mercaptoethanol (β-ME), and the samples were denatured at 95°C for 10 minutes. Proteins were separated by sodium dodecyl sulfate-polyacrylamide gel electrophoresis (SDS-PAGE) and transferred to an Immobilon®-P membrane (Merck Millipore, Burlington, Massachusetts, United States) for 30 minutes using Towbin's transfer buffer in a semi-dry transfer system. The membrane was blocked in Tris-buffered saline (TBS) containing 5% skimmed milk and 0.1% Tween-20 for one hour at 37°C. Membranes were then probed with primary antibodies diluted 1:1000 in blocking buffer overnight at 4°C under continuous agitation. A 20S proteasome β5 mouse monoclonal antibody was used for proteasome detection (sc-393931, Santa Cruz Biotechnology, Dallas, Texas, United States). Normalization was performed using a mouse monoclonal β-actin antibody (sc-47778, Santa Cruz Biotechnology, Dallas, Texas, United States). An anti-mouse horseradish peroxidase (HRP)-linked IgG (7076S, Cell Signaling Technology, Danvers, Massachusetts, United States) was used as a secondary antibody. The blots were subsequently incubated with the appropriate secondary antibodies (anti-rabbit IgG antibody, CST# 7074, or anti-mouse IgG antibody, CST# 7076; both by Cell Signaling Technology, Danvers, Massachusetts, United States) (diluted 1:2000 in blocking buffer) coupled to HRP, and bands were detected with the SuperSignal™ West Femto Maximum Sensitivity Substrate (#34096, Thermo Fisher Scientific, Waltham, Massachusetts, United States), according to the manufacturer's instructions. Quantitative estimation of band size and intensity was performed through the analysis of digital images using ImageJ (National Institutes of Health, Bethesda, Maryland, United States).

Immunofluorescence confocal microscopy

Isolated BMMCs were suspended in phosphate-buffered saline (PBS) solution and left to settle on glass coverslips (MGF slides, Grünwald, Germany) for 45 minutes at 37°C. After this interval, they were gently rinsed with PBS and fixed in a 4% paraformaldehyde aqueous solution for 15 minutes, at room temperature. Subsequently, they were rinsed three times with PBS, permeabilized for 15 minutes in PBS containing 0.1% Triton X-100, and blocked in PBS containing 5% skimmed milk for one hour at room temperature. Cells were then incubated for one hour at room temperature with the primary antibody (20S proteasome β5 subunit antibody (#sc-393931, Santa Cruz Biotechnology, Dallas, Texas, United States)) diluted 1:500 in permeabilization buffer. After thoroughly rinsing with PBS Tween-20, the coverslips were incubated with secondary antibodies (goat anti-mouse IgG (H+L) cross-adsorbed secondary antibody (Alexa Fluor™ 488) (#A-11001, Invitrogen, Waltham, Massachusetts, United States)) diluted 1:500 in permeabilization buffer. After rinsing three times in PBS, cells were mounted using Mowiol 4-88® (Sigma-Aldrich, St. Louis, Missouri, United States).

Flow cytometry

Isolated PBMCs and BMMCs were stained with 2',7'-dichlorodihydrofluorescein diacetate (H_2_DCFDA) (#D399, Invitrogen, Waltham, Massachusetts, United States) for 20 minutes at 37°C to estimate ROS and with the LIVE/DEAD™ Cytotoxicity Dye (Thermo Fisher Scientific, Waltham, Massachusetts, United States) to assess viability. The samples were analyzed with a FACSCalibur™ cytometer (BD Biosciences, Franklin Lakes, New Jersey, United States). For each sample, 200,000 ungated events were acquired.

## Results

HR-MDS patients exhibited higher ChT-L activity and increased β5 subunit expression

**Figure 1:**
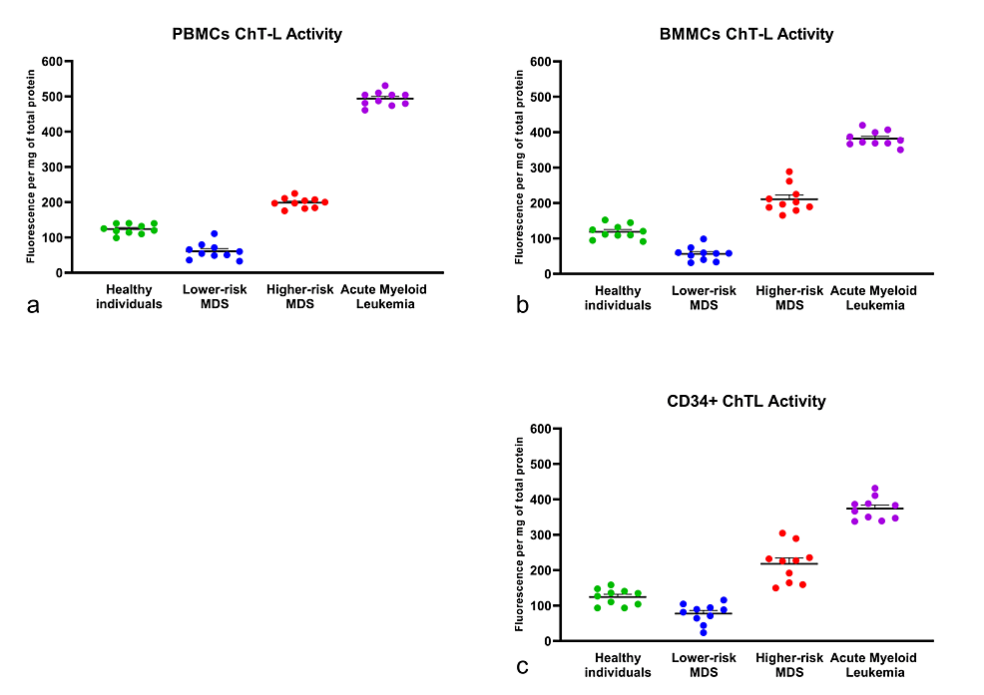
ChT-L activity of hematopoietic cells. Determination of ChT-L activity of (a) PBMCs, (b) BMMCs, and (c) CD34+ cell population isolated from BMMCs. The cell lysates were analyzed with fluorometry, and the results are presented as fluorescence emitted per mg of total protein. The higher-risk group exhibits increased proteasome activity in PBMCs, BMMCs, and CD34+ cells compared to healthy individuals (p<0.0001) as well as the lower-risk group (p<0.0001). The lower-risk group exhibited ChT-L activity even lower than that of healthy individuals (p<0.01), while AML patients were observed to have increased ChT-L activity compared to all groups (p<0.0001). Each sample measurement was conducted in triplicate. ChT-L: chymotrypsin-like; PBMCs: peripheral blood mononuclear cells; BMMCs: bone marrow mononuclear cells

To investigate PPA in MDS patients, we measured ChT-L activity on both PBMCs and BMMCs, as well as on the CD34+ cell population. As controls, we used peripheral blood and bone marrow samples from age-matched healthy subjects. We also collected blood and bone marrow aspirates from patients with AML as positive controls for elevated PPA. Statistical analysis of the obtained values showed that the LR-MDS group exhibited significantly lower ChT-L activity in peripheral blood as compared to the HR-MDS group (Figure 1). Consistently, similar results were also obtained from bone marrow aspirates and from positively selected CD34+ cells of the same patients (Figure 1).

**Figure 2:**
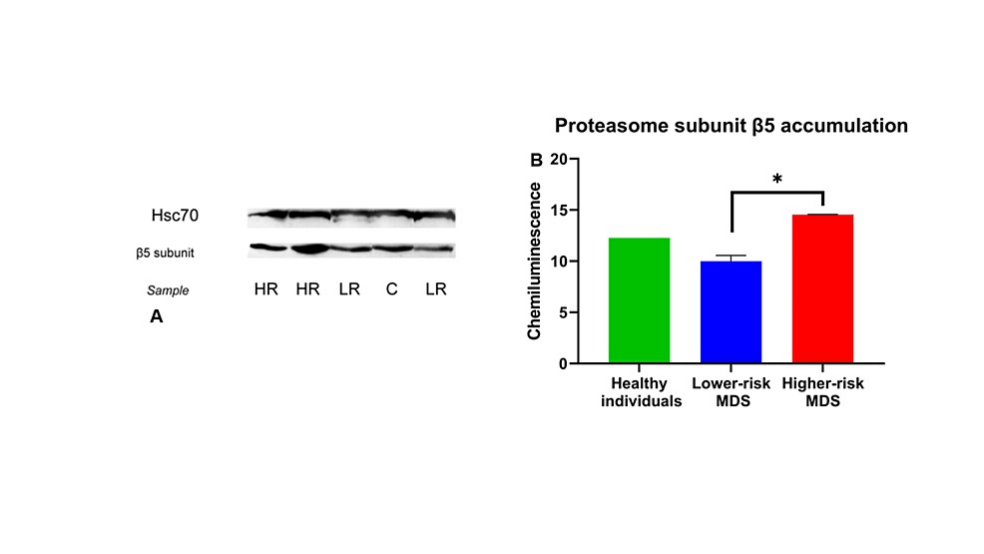
Western blot analysis of the proteasome β5 subunit of hematopoietic cells. The samples were loaded onto 12% polyacrylamide gels and analyzed with SDS-PAGE. Primary antibodies for β5 and for Hsc70 (as a reference gene) were used, and the blots were developed on autoradiography film using the SuperSignal™ West Pico enhanced chemiluminescence (ECL) system (a). The results indicated that the lower-risk group had similar accumulation levels of β5 compared to healthy individuals since no statistically significant changes were observed (b). The higher-risk group exhibited elevated β5 protein levels compared to both healthy individuals (p<0.01) and the lower-risk group (p<0.01). The experiments were performed in triplicate, and for the normalization of the data, the total protein concentration was determined with the Bradford assay. The data analysis was performed using ImageJ and Microsoft Office Excel (Microsoft Corporation, Redmond, Washington, United States). SDS-PAGE: sodium dodecyl sulfate-polyacrylamide gel electrophoresis

Notably, the ChT-L activity of LR-MDS patients was also lower compared to that of healthy subjects. This finding is also consistent with previous observations, according to which CD34+ progenitor marrow cells from MDS patients have reduced PPA compared to the same cell populations of healthy individuals [18]. To evaluate whether a potentially altered β5 subunit expression was responsible for the decreased proteasome ChT-L activity in the LR-MDS patient group, we determined the expression of the β5 subunit in both (LR- and HR-MDS) groups, by Western blot analysis (Figure 2).

**Figure 3:**
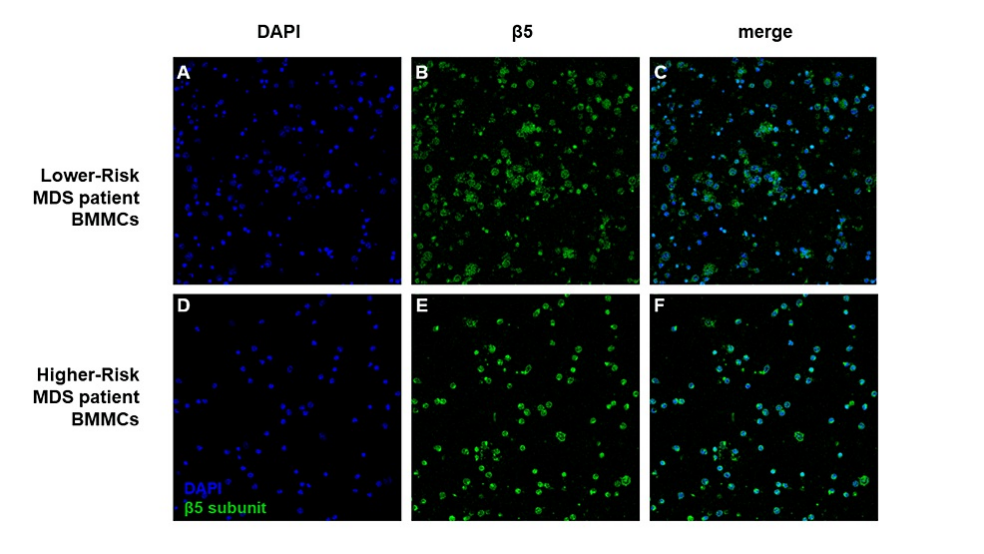
Immunocytochemical staining of the proteasome β5 subunit of hematopoietic cells. BMMCs were attached to glass coverslips, and after fixation and permeabilization, an antibody for β5 was used, as well as DAPI as a nuclear marker. The samples were viewed using a Leica TCS SP8 confocal imaging system (x400 magnification) (Leica Microsystems, Wetzlar, Germany), and the photographs were analyzed using the LAS X software (Leica Microsystems, Wetzlar, Germany). The BMMCs of LR-MDS (a-c) exhibited decreased β5 accumulation levels compared to the HR-MDS group (d-f), while the distribution inside the cell remained mainly cytosolic (c, f). These results complement the Western blot data, verifying the proteasome presence as well as the uniformity of β5 subunit expression among isolated BMMCs. BMMCs: bone marrow mononuclear cells; DAPI: 4′,6-diamidino-2-phenylindole; LR-MDS: lower-risk patients; HR-MDS: higher-risk patients

Overall, a higher expression rate of the proteasome β5 subunit was noticed in HR-MDS patients in both PBMCs and BMMCs. In addition, we investigated the β5 subunit localization inside the BMMCs of lower- and higher-risk patients using confocal microscopy, and we observed uniformity among them. The β5 subunit was observed primarily in the cytoplasm, and the higher expression levels of it inside the BMMCs of higher-risk patients became evident due to the increased fluorescence signal, a result supporting our Western blot findings (Figure 3).

These findings indicate that the increased PPA, which we found in the HR-MDS patient population, resulted from an increased expression of the proteasome β5 subunit. In contrast, no significant difference in the expression levels of the proteasome β5 subunit was observed in either the PBMCs or the BMMCs between healthy subjects and patients in the LR-MDS group. This finding is consistent with the hypothesis that the β5 subunit, produced in the LR-MDS patients, may not be functional, potentially due to the high levels of intracellular oxidative stress that are observed in this group of patients.

HR-MDS patients exhibited lower intracellular ROS levels, compared to the lower-risk group

**Figure 4:**
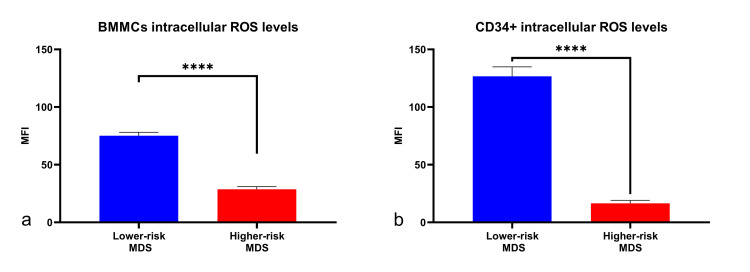
ROS generation in hematopoietic cells of patients with myelodysplastic syndromes was measured using H2DCFDA with flow cytometry. ROS levels were found to be higher in the low-risk group, in both (a) BMMCs and (b) the CD34+ population. The assay was performed using identical cell numbers, assay temperature, and staining time, and the samples were analyzed with a FACSCalibur cell cytometer. To distinguish between live and necrotic cells, the LIVE/DEAD kit was used, and to measure ROS levels, the substance H_2_DCFDA reacts with OH· radicals. The CD34+ population was gated using an anti-CD34 mouse IgG antibody conjugated with Alexa Fluor 647 (Thermo Fisher Scientific, Waltham, Massachusetts, United States), and the data were analyzed using FlowJo (BD Biosciences, Franklin Lakes, New Jersey, United States). ROS: reactive oxygen species; H_^2^_DCFDA: 2',7'-dichlorodihydrofluorescein diacetate; BMMCs: bone marrow mononuclear cells

After evaluating PPA and performing quantification assays, the oxidative stress levels were assessed by estimating the total amount of intracellular ROS generation. The BMMCs, and particularly the positively selected CD34+ cell subpopulation, were assessed for this purpose since the detrimental effects of the free radicals are well-known to induce mutagenesis and malfunction in the bone marrow progenitor cells of MDS patients, which are later released into the peripheral circulation. The oxidative stress of plasma and of the total PBMCs was not assessed in this study since it was expected to be elevated to high levels as a result of the frequent RBC transfusions that many MDS patients were receiving, whereas the purpose of this study was to evaluate the preexisting cell phenotype inside the bone marrow cells as a marker of impaired proteostasis. Interestingly, intracellular ROS levels were found to be decreased in patients with a higher IPSS score as compared to the lower-risk group, both in BMMCs and in the CD34+ progenitor cell population (Figure 4). The BMMCs of HR-MDS patients exhibited half the amount of intracellular ROS levels compared to lower-risk patients, while the CD34+ subpopulation had almost eight times lower ROS levels.

Transfusion dependence affects both ChT-L activity and intracellular ROS levels

**Figure 5:**
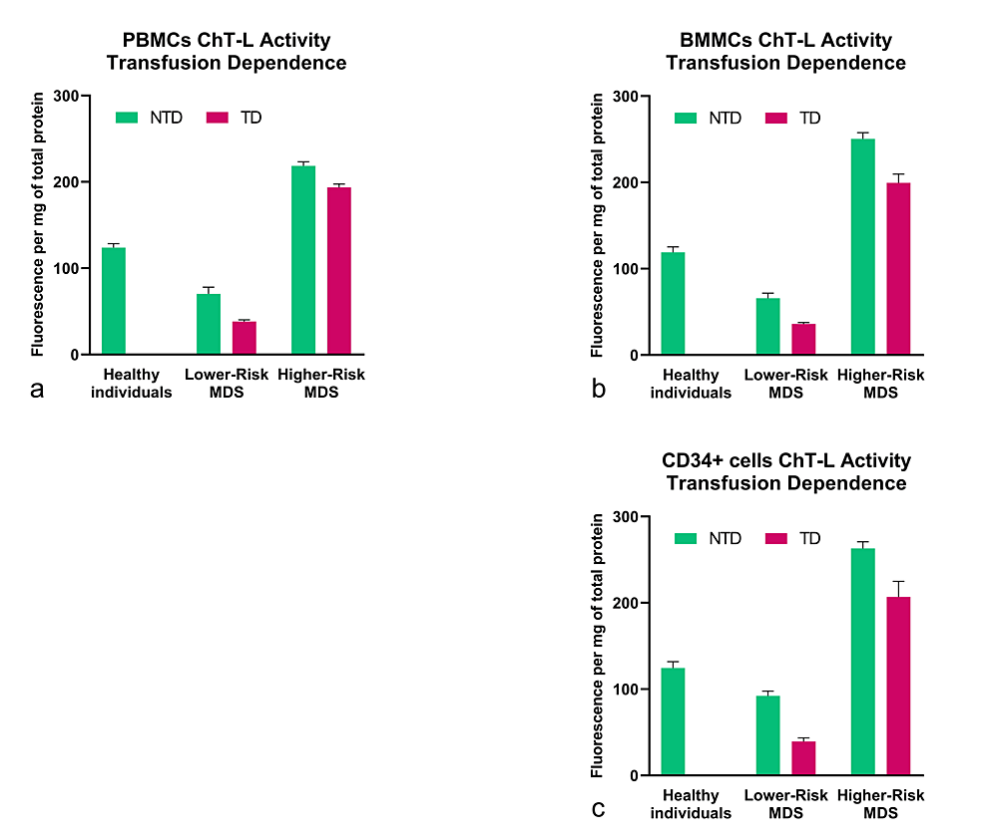
ChT-L activity is correlated to transfusion dependence. Our data indicated that TD patients exhibit lower ChT-L levels compared to NTD patients in both groups (p<0.0001). The same phenomenon was documented in (a) peripheral blood mononuclear cells (p<0.001), (b) bone marrow mononuclear cells (p<0.0001), and (c) the hematopoietic CD34+ cells (p<0.0001). The results were analyzed with Prism™ 8 using multiple t-tests. ChT-L: chymotrypsin-like; TD: transfusion-dependent; NTD: non-transfusion-dependent

Besides the basic prognostic categorization of the patients into a lower- and a higher-risk group, we also investigated whether there was an association between transfusion dependency and the studied parameters. The data were analyzed with Prism 8 (GraphPad, La Jolla, California, United States) software using multiple t-tests. According to our results, transfusion-dependent (TD) patients were observed to have significantly lower PPA in PBMCs, BMMCs, and the CD34+ population (p<0.0001) (Figure 5).

**Figure 6:**
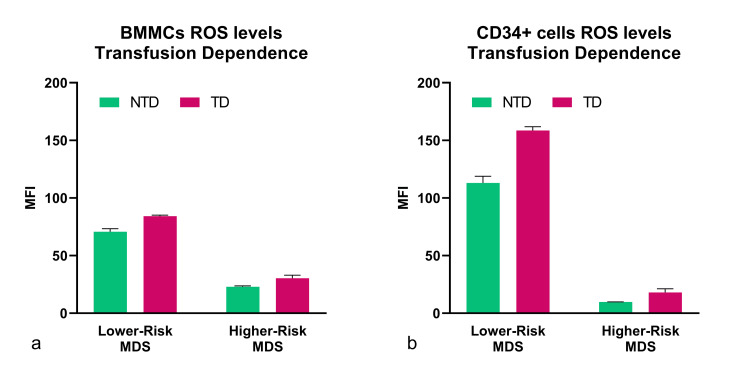
Intracellular ROS levels are correlated to transfusion dependence. Our data showed that TD patients had elevated intracellular ROS content compared to NTD patients in both groups. The phenomenon was documented in (a) bone marrow mononuclear cells (p<0.0001), and the observations in (c) the hematopoietic CD34+ cell population were consistent (p<0.0001). The results were analyzed with Prism™ 8 using multiple t-tests. ROS: reactive oxygen species; TD: transfusion-dependent; NTD: non-transfusion-dependent

ROS levels were also analyzed using the same principle, and an elevation of oxidative stress was documented in BMMCs and CD34+ cells in both patient groups (lower and higher risk) as long as those individuals were TD (Figure 6).

## Discussion

Proteasomal function is responsible for many crucial cellular processes, such as protein metabolism, cell cycle, division, and apoptosis. Inadequacy or impairment of the functional subunits of the proteasome, which are usually observed in LR-MDS, could lead to the accumulation of ROS due to a lack of oxidized protein degradation and dysregulation of cellular homeostasis [22]. Based on the results obtained from this study, targeted therapies, such as PIs, might not be effective in LR-MDS patients. The activity of the proteasome was found to be substantially low in these patients, and thus, the PI drug effect would be negligible. On the other hand, the high ChT-L activity that we detected in HR-MDS patients appears to be similar to that reported in other hematological malignancies, such as AML and multiple myeloma [23,24]. Our findings for HR-MDS are aligned with those reported from other studies, in which ChT-L activity, measured in the plasma of patients with AML and advanced-stage MDS, was found to be highly elevated [25]. Results from parallel clinical trials have shown that the addition of PIs, such as bortezomib, to low-dose chemotherapy in HR-MDS patients may improve the results obtained with the use of chemotherapy alone, particularly in patients with unfavorable karyotypes [26].

To clarify the causality of the decreased PPA detected among patients with LR-MDS, we investigated the hypothesis that the high apoptotic rate observed in these patients might be associated with the high ROS levels [26]. As a confirmation of this hypothesis, we found significantly higher intracellular ROS levels in the lower-risk patient group compared to the higher-risk group, a result that might explain the defective UPS function reported among LR-MDS patients. Several in vitro studies have shown that in the hematopoietic stem cells of patients with MDS and AML, the constitutional ROS equilibrium is often defective [20,27]. An interesting in vitro study has demonstrated that various molecules involved in cell metabolism are considered crucial regulators of ROS levels and, simultaneously, of various redox sensor molecules [28]. In other words, an oxidative abnormality, supervening in one or more of the different signals in which these molecules are involved (mostly present in solid tumors), may alter the key metabolic pathways related to stem cell fate, changing processes that regulate cell cycle progression, apoptosis, quiescence, and/or differentiation [28].

Furthermore, the observation that in TD MDS patients, irrespective of the risk group, PPA is lower and ROS levels are higher compared to NTD patients implies a potential key role of iron overload. Excess intracellular iron is generally accumulated as ferritin and hemosiderin [29]. When the level of labile cellular iron (LCI), the free iron form, increases, it is responsible for potentially dangerous free radical-generating reactions with the production of hydroxyl radicals, ultimately leading to cellular death and consequent tissue damage [30]. Essentially, when serum transferrin saturation exceeds the level of 70-75%, non-transferrin-bound iron (NTBI) and its subcomponent labile plasma iron (LPI) appear in the serum [31]. LPI is capable of entering the cells through alternative channels (other than transferrin receptor canals), increasing LCI levels. When ROS production exceeds the neutralizing capacity of the antioxidant enzyme systems, an excessive accumulation of ROS occurs, leading to increased intracellular oxidative stress [28]. Proteasome is a highly dynamic protein complex capable of adjusting its proteolytic activity depending on the needs of the cell. According to the model of oxidative stress-dependent regulation of proteasomes, in the early phase of cellular response to oxidative insult, various changes occur to modulate PPA in order to promote the degradation of oxidized proteins and limit the damage of oxidative stress. In cases of persistent oxidative insults such as long-lasting iron overload, proteasomes disassemble, PPA diminishes, and de novo proteasome synthesis is activated [32]. Therefore, the working hypothesis is that cellular toxicity may not mainly represent a direct result of stored iron but rather emerges from the disruption of the dynamic balance that exists between the storage and functional iron pools [33]. Thus, iron chelation therapy may not only improve the hematopoietic insufficiency of MDS patients by reducing the rate of apoptosis of the dysplastic progenitors in the marrow, but it could also delay the progression toward acute leukemia [34]. Indeed, Malcovati et al. have described that leukemia-free survival was lower in MDS patients with higher ferritin levels and in those requiring high blood transfusion rates [35].

As a general rule, MDS patients should be treated following different treatment algorithms according to their risk group. Based on our observations and findings, the beneficial effect of HMAs and low-dose chemotherapy, when combined with PIs such as bortezomib, is indisputable in the higher-risk patient group and in AML patients [36]. In contrast to other hematological malignancies or solid tumors, LR-MDS displays a lower PPA, and this observation could lead to reconsideration of the potential therapeutic targets. In this patient group, only one exploratory clinical trial of bortezomib has been performed, and the results showed modest clinical activity in these patients [17]. Evaluation of oxidative stress and proteasome activity could be used as screening tools for prognosis and the application of new therapeutic options. Several studies have reported the potential usefulness of antioxidant agents or iron chelation therapy that can act as antioxidants for MDS patients [37,38]. In conclusion, we suggest that, beyond targeted therapies applied for specific cytogenetic or molecular abnormalities, emphasis should also be placed on reducing intracellular oxidative stress that could lead to the restoration of proteasome activity and the subsequent reduction of apoptosis.

## Conclusions

Based on our results, targeted therapies such as PIs could not be effective for LR-MDS patients. PPA was found to be significantly lower in these patients, and the drug effectiveness is anticipated to be negligible. In addition, the high ROS levels, usually found in LR-MDS patients, can be associated with their high apoptotic potential and the multiple cytopenias that these patients usually manifest. Additionally, transfusion dependence was found to further increase intracellular ROS levels and simultaneously decrease PPA in both patient groups. Evaluation of oxidative stress and proteasome activity could be used as screening tools for prognosis and possibly for the application of new therapeutic options.

## Appendices

Abbreviations

AML                Acute myeloid leukemia

BMMCs          Bone marrow mononuclear cells

CD34+           Cluster of differentiation 34 positive

ChT-L             Chymotrypsin-like

C-L                 Caspase-like

ESA                Erythropoiesis-stimulating agents

H_2_DCFDA       2',7'-Dichlorodihydrofluorescein diacetate

HEPES           4-(2-Hydroxyethyl)-1-piperazineethanesulfonic acid

HMAs            Hypomethylating agents

HR-MDS        Higher-risk myelodysplastic syndrome

HRP                Horseradish peroxidase

ICT                 Iron chelation treatment

IPSS               International Prognostic Scoring System

KCl                 Potassium chloride

LCI                 Labile cellular iron

LLVY-AMC    Leucine-leucine-valine-tyrosine-7-amino-4-methylcoumarin

LPI                 Labile plasma iron

LR-MDS        Lower-risk myelodysplastic syndrome

MACS            Magnetic-activated cell sorting

MDS              Myelodysplastic syndrome

MgCl_2_            Magnesium chloride

NTD               Non-transfusion-dependent

NTBI              Non-transferrin-bound iron

PBMCs         Peripheral blood mononuclear cells

PBS               Phosphate-buffered saline

PIs                 Proteasome inhibitors

PPA               Proteasome proteolytic activity

PSMB5         Proteasome subunit beta-5

RIPA              Radioimmunoprecipitation assay

RBCs             Red blood cells

ROS               Reactive oxygen species

SDS-PAGE    Sodium dodecyl sulfate-polyacrylamide gel electrophoresis

TBS               Tris-buffered saline

TD                 Transfusion-dependent

T-L                Trypsin-like

UPS              Ubiquitin-proteasome system

WB               Western blot
